# Biomimetic Cucurbitacin B-Polydopamine Nanoparticles for Synergistic Chemo-Photothermal Therapy of Breast Cancer

**DOI:** 10.3389/fbioe.2022.841186

**Published:** 2022-02-09

**Authors:** Junke Leng, Xiaofeng Dai, Xiao Cheng, Hao Zhou, Dong Wang, Jing Zhao, Kun Ma, Changhao Cui, Li Wang, Zhaoming Guo

**Affiliations:** ^1^ School of Life and Pharmaceutical Sciences, Dalian University of Technology, Panjin, China; ^2^ School of Food and Environment, Dalian University of Technology, Panjin, China; ^3^ Panjin People’s Hospital, Panjin, China

**Keywords:** targeted drug delivery, photothermal therapy, cucurbitacin B, biomimetic, synergistic treatment

## Abstract

Breast cancer is the most common malignant tumor in women. Researchers have found that the combined use of multiple methods to treat tumors is a promising strategy. Here, we have developed a biomimetic nano-platform PDA@MB for tumor targeted photothermal therapy (PTT) combined with chemotherapy. The 4T1 cell membrane loaded with cucurbitacin B (CuB) was used to coat polydopamine (PDA) nanoparticles, which gave PDA@MB nanoparticles the ability to target tumors and escape immune cells from phagocytosis. PDA@MB showed excellent photothermal performance including high photothermal conversion efficiency and photostability, and exhibited outstanding *in vitro* PTT effect under NIR laser irradiation. The high temperature ruptured the PDA@MB membrane to release CuB, which changed the tumor hypoxic environment, down-regulated the FAK/MMP signaling pathway, and significantly inhibited the metastasis and proliferation of tumor cells. The results of *in vivo* experiments indicated that the tumor growth of the 4T1 mouse tumor model was significantly inhibited. Additionally, toxicity studies showed that PDA@MB had good biocompatibility and safety. In conclusion, this study provides a promising chemo-photothermal therapy (CPT) nano-platform for precise and effective breast cancer therapy.

## 1 Introduction

Female breast cancer is the most common cancer, accounting for 11.7% of the overall cancer incidence (Sung et al., 2021). Metastasis is the main reason for its refractory (Siegel et al., 2019). Cucurbitacin B (CuB) is a tetracyclic triterpenoid compound and the most abundant member of the cucurbitacin family. There are literatures showing that CuB and its analogues have pharmacological effects on inflammation, especially cancer (Chen et al., 2005; Chen et al., 2012). CuB inhibits the migration and invasion of breast cancer by down-regulating the FAK/MMP signaling pathway (Liang et al., 2019). It is closely related to the extracellular matrix degradation of matrix metalloproteinases (MMP_S_) to avoid primary tumors and form focal adhesions (Sinha et al., 2016). In addition, CuB mediates rapid and large-scale ROS production in cancer cells to promote anti-cancer and anti-metastatic activities (Luo et al., 2018). However, free chemotherapeutics have serious side effects due to oxidative stress in non-targeted tissues (Chen et al., 2007). Therefore, it is necessary to accurately deliver chemotherapeutic drugs to target sites to reduce toxic side effects. Nano drug delivery systems have received great attention, because it can improve the effect of targeting tumors and greatly reduce side effects (Shi et al., 2017; Dadwal et al., 2018). The precise delivery of drugs to the tumor site through nano-carriers will greatly increase the possibility of curing the tumor.

Recently, photothermal therapy (PTT) with high efficiency and minimal invasiveness is considered as a new promising option for cancer treatment (Gai et al., 2018; Wang et al., 2018; Cai et al., 2019; Zhang et al., 2019). Compared with traditional cancer treatments, PTT has fewer side effects due to the high temporal and spatial control of local heat (Chen et al., 2016). Some nanocarriers are also photosensitizers. Dopamine (DOPA) is an important endogenous neurotransmitter in the nervous system. It is mainly used to help cells transmit pulses and plays a key role in controlling the excitability of the brain. Polydopamine (PDA) is formed by polymerization of DOPA. As a photosensitizer, compared with Au-, Ag-, and Pd-based novel metal nanoparticles, PDA can effectively convert near-infrared light into heat, and has good biocompatibility and biodegradability (Liu et al., 2013; Li et al., 2018). As researchers learn more about the interactions between nanoparticles (NPs) and biological systems, the functionalization of materials is necessary. Some observations indicate that PEG-modified NPs can attenuate non-specific interactions with biological systems. However, PEG can trigger another type of immune response, leading to the production of anti-PEG antibodies, which may bring about adverse effects on the multiple administration of NPs and their therapeutic potential (Neun et al., 2018; Mohamed et al., 2019). Therefore, advanced NP_S_ should be designed to overcome physical and biological obstacles and bridge the gap between synthetic NP_S_ and biological entities. Due to the challenges of synthesizing functionalized nanoparticles, the development of biomimetic nanotechnology by coating natural-derived biofilms for surface modification has aroused interest in nanomedicine (Zhen et al., 2019). Tumor cell membrane coating technology provides new ideas for nanoparticle drug delivery systems. This technology provides a simple top-down method that can directly replicate the highly complex functions of the cell membrane surface (Li et al., 2017; Jiang et al., 2019). The membrane-coated nanoparticles themselves mimic the properties of their membrane-derived source cells, such as excellent accidental targeting and immune escape ability, and reduce the side effects of drugs and nanomaterials (Fang et al., 2017; Tang et al., 2017).

**SCHEME 1:**
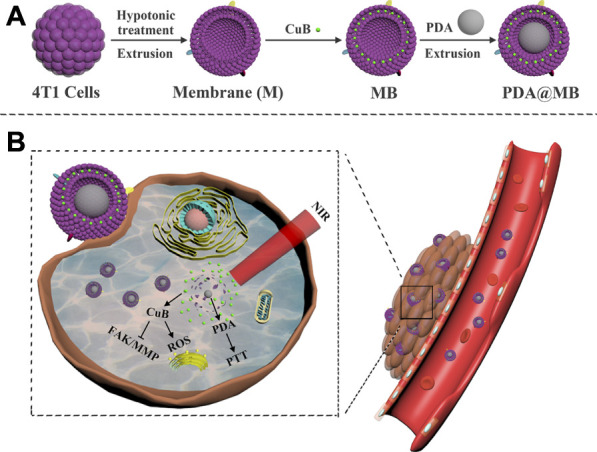
**(A)** Schematic illustration of the preparation of PDA@MB. **(B)** Schematic illustration of PDA@MB CPT platform for tumor combined therapy.

In this study, we designed a bionic nano-delivery system PDA@MB for precise targeted tumor chemo-photothermal therapy (CPT), which used PDA NPs as the core, CuB as the model drug, and tumor cell membrane as the outer coating. As shown inScheme 1A, the mouse breast cancer cells (4T1 cells) were used to obtain the membrane vesicles by hypotonic lysis and extrusion. Then, the anticancer drug CuB was embedded in the cancer cell membrane to obtain MB. Finally, PDA NPs and MB were co-extruded to obtain PDA@MB. The cell membrane coating improved the biocompatibility of the nanoparticles, and endowed the nanoparticles with homotype targeting ability and immune escape ability. Because of the EPR effect (Golombek et al., 2018) and active targeting, PDA@MB could accumulated at the tumor site after intravenous injection and administration (Scheme 1B). Under the irradiation of laser at 808 nm, PDA converted light into heat energy, and killed tumor cells through PTT. At the same time, the high temperature ruptured the PDA@MB membrane to release CuB, which mediated the rapid mass production of ROS in cells, down-regulated the FAK/MMP signaling pathway, and inhibited tumor cell adhesion, metastasis and proliferation. *In vivo* and *in vitro* experiments showed that PDA@MB had a good therapeutic effect on breast cancer mouse models, indicating that PDA@MB could be used as a CPT platform to achieve effective tumor combined therapy.

## 2 Materials and Methods

### 2.1 Materials

Dopamine hydrochloride was purchased from McLean (Shanghai, China). Cucurbitacin B was provided by Chengdu Biopurify Phytochemicals Ltd. (Chengdu, China). 4′,6-Diamidino-2-phenylindole (DAPI), RIPA lysis buffer, BCA assay kits, Rhodamine B and annexin V-FITC/PI cell apoptosis kit were obtained from Solarbio (Beijing, China). DCFH-DA and Cell Counting Kit-8 (CCK-8) were purchased from Beyotime Institute of Biotechnology (China). Foetal bovine serum (FBS), RPMI modified medium, trypsin-EDTA and penicillin-streptomycin were purchased from HyClone. All antibodies were obtained from ABclonal.

### 2.2 Cell Culture

The mouse breast cancer cell line (4T1) was cultured in RPMI 1640 medium containing 10% fetal bovine serum, 100 U/ml penicillin and 100/U ml streptomycin. RAW264.7 cells and 293T cells were cultured in DMEM medium containing 10% fetal bovine serum, 100 U/ml penicillin and 100/U ml streptomycin. Both types of cells were cultured in an incubator at 37°C under an atmosphere of 5% CO_2_ and 90% relative humidity.

### 2.3 Preparation of PDA@MB

#### 2.3.1 Synthesis of Polydopamine

The PDA NPs were prepared by oxidation method. In short, absolute ethanol (40 ml), deionized water (90 ml) and ammonia (4 ml) were mixed and stirred at room temperature for 30 min. Then dopamine hydrochloride solution (0.5 g/ml, 10 ml) was added dropwise to the above solution. After 24 h of reaction, the obtained suspension was centrifuged and washed with deionized water three times to obtain PDA NPs.

#### 2.3.2 Preparation of 4T1 Cell Membrane Derivation

Adherent 4T1 cells were digested with trypsin, and lysed overnight in a buffer at 4°C. The buffer was a mixture of NaHCO_3_ (1 mM), trypsin-EDTA (.2 mM), and PMSF (1 mM). Then, the 4T1 cell suspension was homogenized at 20,000 rpm for 1 min. The homogenized solution was centrifuged at 3,500 g for 5 min and 10,000 g for 10 min at 4°C. Finally, the supernatant was centrifuged at 100,000 g for 1 h. The membrane pellet was washed and resuspended in PBS for storage at 4°C.

#### 2.3.3 Preparation of PDA@MB

4T1 membrane (2 mg) and cucurbitacin B (1 mg) were dissolved in PBS (10 ml) and DMSO (1 ml), respectively. The above mixture was vigorously stirred for 1 h. The 4T1 membrane with cucurbitacin B (MB) was obtained after centrifugation and washing. In order to obtain MB vesicles, the extracted MB was extruded repeatedly through a 400 nm polycarbonate microporous filter membrane for 15 times by a liposome extruder. Then, PDA NPs and MB vesicles were repeatedly co-extruded through a 200 nm polycarbonate microporous membrane for 15 times to obtain PDA@MB.

### 2.4 Characterization

The morphology of nanoparticles was characterized by transmission electron microscopy (TEM) (FEI Tecnai G2 F30). The hydrated particle size and zeta potential of PDA and PDA@MB were measured by the dynamic light scattering (DLS) analysis using Malvern Zetasizer Nano ZS (Malvern, UK). FT-IR spectrum was measured by Fourier transform infrared spectrometer (FT-IR, Nicolet iN10 MX & iS10, ThermoFish) with KBr pellets. The optical absorbance was measured by a UV–vis spectrophotometer (Theromo Scientific Instrument Co., Ltd.). The irradiation was performed using a NIR laser with a center wavelength of 808 nm (Changchun New Industries Optoelectronics Tech. Co., Ltd.).

### 2.5 Protein Dection of PDA@MB

The sodium dodecyl sulfate-polyacrylamide gel electrophoresis (SDS-PAGE) was used to detect the membrane protein of PDA@MB. The 4T1 cell membrane protein and the membrane protein of PDA@MB were extracted with radio immunoprecipitation assay (RIPA) lysate. The concentration of the extracted protein was determined by the BCA kit. The protein sample and 5×loading buffer were boiled in a 100°C water bath for 5 min and cooled to room temperature. The above sample (10 μg) was added to the well of 10% concentrated gel. The electrophoresis was stopped when the bromophenol blue indicator moved to the bottom of the gel. Finally, the SDS-PAGE gel was stained with Coomassie brilliant blue R250 for 30 min and photographed after 15 h of decolorization.

### 2.6 *In Vitro* Drug Release

In order to determine the release of CuB, PDA@MB (150 μg/ml, laser or non-laser irradiation) was dissolved in PBS (pH = 5.0 or 7.4, 1 ml) and placed in a dialysis bag (MW = 14,000). Then, the dialysis bag was slowly shaken in a PBS (10 ml) system at 37°C (100 r/min). PBS solution (1 ml) was withdrawn at predetermined time points and fresh PBS solution (1 ml) was added after each sampling. The amount of released CuB was measured by UV-vis spectrophotometer.

### 2.7 Photothermal Performance Measurement of PDA@MB

In order to evaluate the photothermal performance of PDA@MB, PDA@MB solution of different concentrations (0, 20, 40, 80, 100, 120, and 140 μg/ml, 1 ml) were irradiated for 10 min with the NIR laser (808 nm, 2 W cm^−2^). A digital thermocouple was utilized to record the data every 1 min and an infrared camera was used to photograph the infrared thermal images every 2 min. To calculate the photothermal conversion efficiency (η) of PDA@MB, PDA@MB solution (100 μg/ml, 1 ml) and deionized water were irradiated with laser (808 nm, 2 W cm^−2^) for 10 min and then the laser irradiator was turned off to allow the solution to cool naturally to ambient temperature. The heating data and cooling data were recorded every 1 min, and the temperature-time diagram was plotted.

### 2.8 Toxicity and Hemolysis Assay

CCK-8 method was used to evaluate the cytotoxicity of PDA and PDA@MB. 293T cells (5,000 cells/well) were seeded in a 96-well plate and cultured overnight. The culture medium was drawn out. Then, cell culture medium containing different concentrations of PDA or PDA@MB (0, 20, 40, 60, 80 and 100 μg/ml) was added to each well and incubated for 24 h. CCK-8 (10 μl) was added to each well and incubated at 37°C for 1 h. Finally, a microplate reader was used to measure the absorbance value at 450 nm.

The blood compatibility of PDA and PDA@MB was determined by hemolysis test. Blood was collected from the venous plexus of the fundus of the mouse and placed in an anticoagulant tube. The blood cells were washed 3 times with PBS and centrifuged at 1,000 r/min at 4°C. Then, blood cells (20 μl) were added to different concentrations of PDA and PDA@MB (20, 40, 60, 80, 100 μg/ml) and slowly shaken (100 r/min) in a 37°C shaker. Photographs were taken after 4 h of incubation. The supernatant was sucked into a 96-well plate and a microplate reader was used to measure the OD value at 540 nm. The OD values of deionized water and PBS were positive and negative controls to calculate the hemolysis rate of PDA and PDA@MB nanoparticles. The formula is as follows:

$$Hemolysis\;rate\;\left(\%\right)=\frac{A_{sample}-A_{negative}\;}{A_{positive}-A_{negative}}\times 100\%$$

Where A_sample_ refers to the absorbance of the sample at 540 nm. A_positive_ and A_negative_ represent the absorbance of the positive control and the negative control at 540 nm, respectively.

### 2.9 Detection of Intracellular ROS

Confocal laser scanning microscopy (CLSM, Heidelberg, Germany) and flow cytometry (Becton Dickinson FACSCalibur, USA) were used to detect the production of ROS in cells by DCFH-DA active oxygen analysis kit. For CLSM imaging experiments, 4T1 cells (10,000 cells/well) were cultured overnight in a 24-well plate. Then, the cells were incubated with PDA or PDA@MB (100 μg/ml, 7.5 μg/ml CμB) for 6 h. After that, the cells were washed with PBS and treated with serum-free medium containing DCFH-DA (10 μM) for 30 min. Subsequently, the cells were irradiated with laser (808 nm, 2 W cm^−2^) for 5 min as the laser irradiation groups. After another .5 h of incubation, the fluorescence intensity of 4T1 cells was observed by CLSM. For flow cytometry analysis, the cells were processed in the same way. Finally, the cells were collected and resuspended in PBS (600 μl) for detection within 30 min.

### 2.10 Western Blotting Analysis

Western blotting was used to analyze the expression of key proteins related to tumor metastasis. In PDA and PDA@MB groups, 4T1 cells were incubated with PDA and PDA@MB (100 μg/ml, 7.5 μg/ml CμB) for 6 h, respectively. After 5 min of laser irradiation (808 nm, 2 W cm^−2^), the cells were put back into the incubator and incubated for another 3 h. The proteins were extracted with RIPA lysate. The concentration of the extracted proteins was determined by the BCA kit. The protein sample and 5×loading buffer were boiled in a 100°C water bath for 5 min and cooled to room temperature. After the proteins were separated by SDS-PAGE electrophoresis, they were transferred to PVDF membrane and blocked with 5% skimmed milk powder at room temperature for 1 h. Then, the PVDF membrane was incubated with the primary antibodies of pFAK, FAK, MMP-9, MMP-2 and GAPDH overnight at 4°C. After washing, the membranes were incubated with appropriate HRP-conjugated secondary antibodies for 1 h. ECL luminescent solution was added to visualize the protein bands.

### 2.11 Cellular Uptake Assay

Rhodamine B (Rho B) was used to label nanoparticles to study the homologous targeting ability of PDA@MB. 4T1 cells (200,000 cells/well) were seeded in a 24-well plate. After 24 h of incubation, Rho-PDA or Rho-PDA@MB (100 μg/ml) were added to the wells and incubated with the cells for 1 h. The cells were fixed with 4% paraformaldehyde. After washing with cold PBS, nucleus dye DAPI was added and incubated with the cells for 15 min. Finally, CLSM was used for imaging. To further quantify the cancer cell targeting efficiency of M-coated nanoparticles, 4T1 cells (500,000 cells/well) were seeded in a 12-well plate and incubated with Rho-PDA or Rho-PDA@MB for 1 h. Then, the cells were washed and resuspended in PBS to monitor the fluorescence signal using a flow cytometer within 30 min.

To assess the phagocytic evasion ability of PDA@MB, RAW264.7 cells were treated with Rho-PDA or Rho-PDA@MB for 1h. CLSM was used to qualitatively observe the uptake of nanoparticles by RAW264.7 cells. The quantification of intracellular fluorescence was performed using a flow cytometer.

### 2.12 *In Vitro* Anti-Tumor Studies

Inverted fluorescence microscope was used for qualitative analysis. 4T1 cells were cultured overnight in a 96-well plate. Different concentrations of PDA@MB (0, 20, 60, 100 μg/ml PDA, 0, 1.5, 4.5, 7.5 μg/ml CμB) were added and incubated with cells for 6 h. After treatment with an NIR laser (808 nm 2 W cm^−2^) for 5 min, the 96-well plate was returned to the cell culture incubator and incubated for 3 h. The live and dead cells were stained with Calcein-AM/PI and fluorescence images were taken with an inverted fluorescence microscope (Leica DMI4000B).

Flow cytometry was used for quantitative analysis. 4T1 cells were incubated with culture medium containing PDA@MB (0, 20, 60 or 100 μg/ml) in a 96-well plate for 6 h. Then, the cells were washed with PBS and irradiated with an NIR laser (808 nm 2 W cm^−2^) for 5 min. After irradiation, the 96-well plate was returned to the cell culture incubator and incubated for 3 h. For flow cytometry analysis, the live and dead cells were stained with Annexin V FITC and PI, respectively.

For the CCK-8 assay, 4T1 cells were seeded in 96-well plates and incubated overnight. Afterwards, the cell culture medium containing PDA@MB (0, 20, 60 or 100 μg/ml) was added to each well and incubated for 6 h. After irradiation, the 96-well plate was returned to the cell culture incubator for 3 h. CCK-8 (10 μl) was added to each well to determine the OD value at 450 nm using a microplate reader (BioTek, USA).

### 2.13 Animal Model

BALB/c female mice (6 weeks old, Liaoning Changsheng biotechnology Co., Ltd.) were used to establish tumor models. 4T1 cells (6 × 10^5^, 100 μl) were subcutaneously injected into the armpits of mice. *In vivo* experiments were started when the tumor volume of mice reached 50 mm^3^. The formula for calculating tumor volume is as follows:

$$Tumor\;volume\;\left(mm^{3}\right)=\frac{W^{2}\times L}{2}$$

Where W and L refers to the shortest diameter and the longest diameter of the tumor, respectively. All animal procedures in this study were approved by the Institutional Animal Care and Use Committee of Dalian University of Technology.

### 2.14 *In Vivo* Combination Therapy Effect

The mice were randomly divided into 8 groups (*n* = 4): (a) PBS; (b) Laser; (c) PDA; (d) PDA + Laser; (e) CuB; (f) PDA@M + Laser; (g) PDA@MB; (h) PDA@MB + Laser; Each mouse was intravenously injected with PBS, PDA, PDA@M, CuB or PDA@MB (10 mg/kg PDA, .15 mg/kg CuB, 100 μl). After 24 h of administration, the tumor sites of mice in groups (b), (d), (f) and (h) were irradiated with laser (808 nm 2 W cm^−2^) for 5 min. The infrared images of the mice in each group were taken at 0, 3 and 5 min. The mice were weighed and the tumor volume was measured every 2 days during the treatment.

### 2.15 Blood Biochemical Analysis and Histopathological Examination

Fresh mouse blood was collected at the end of the experiment for routine blood analysis and biochemical analysis. Then, the main organs (heart, liver, spleen, lung, kidney) of the mice were collected and soaked in a neutral 10% formalin solution. Paraffin sections were prepared and stained with haematoxylin and eosin (H&E). The optical microscope was used to observe and photograph the slides.

### 2.16 Statistical Analysis

All experiments were repeated at least three times, and all results are presented as the mean ± SD. Statistical significance was evaluated using the two-tailed heteroscedastic Student’s t-test (∗*p* < .05; ∗∗*p* < .01; ∗∗∗*p* < .001; ∗∗∗∗*p* < .0001).

## 3 Results and Discussion

### 3.1 Synthesis and Characterization of PDA@MB

**FIGURE 1:**
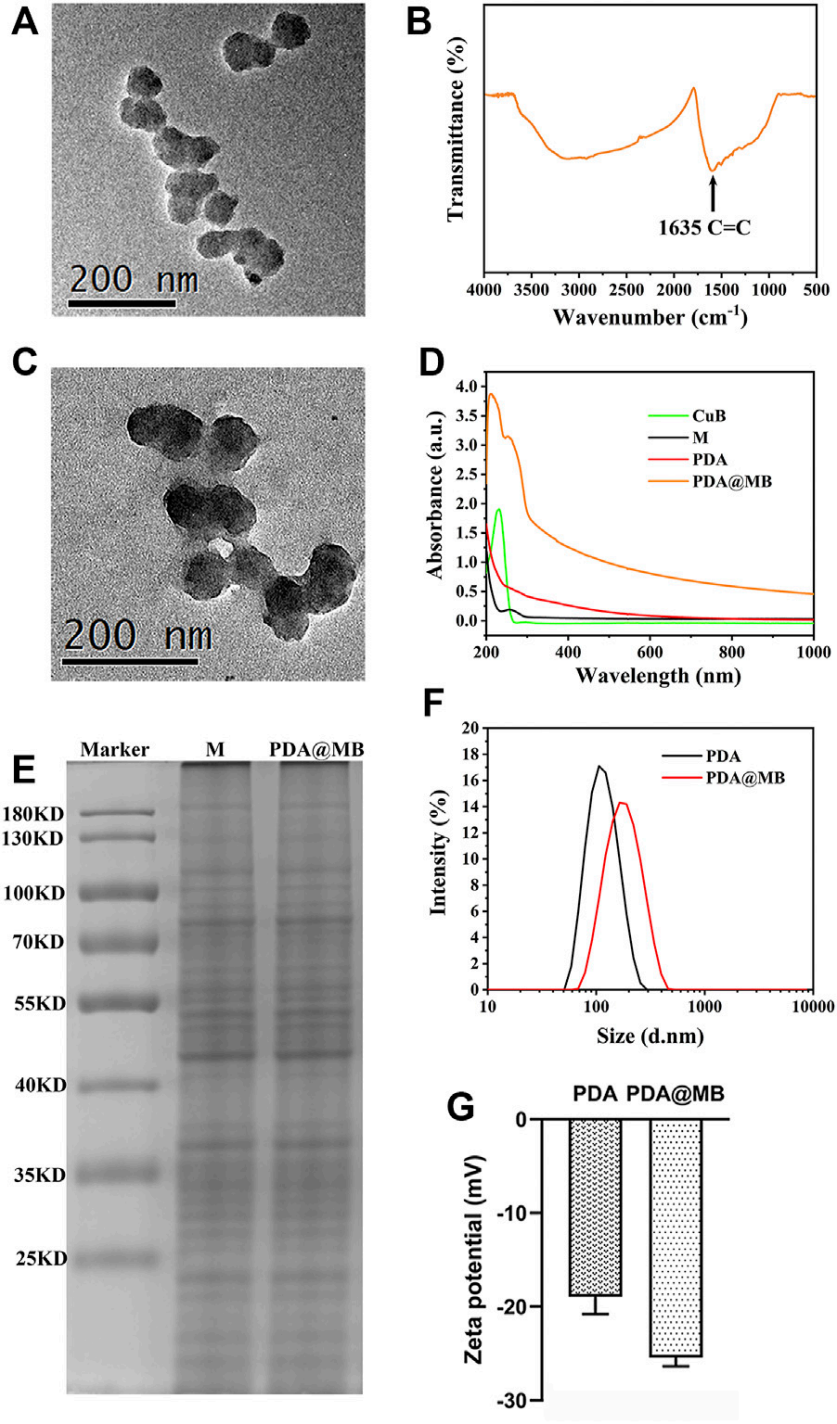
**(A)** TEM image of PDA. The scale bar is 200 nm. **(B)** FT-IR spectroscopy of PDA. **(C)** TEM image of PDA@MB. **(D)** UV-Vis absorption spectra of CuB, 4T1 cell membrane (M), PDA and PDA@MB. **(E)** SDS-PAGE-based protein analyses of 4T1 cell membrane (M) and PDA@MB. **(F)** Hydrodynamic diameter of PDA NPs and PDA@MB. **(G)** Zeta potential of PDA NPs and PDA@MB.

The synthesis procedure of PDA@MB is outlined in Scheme 1A. Briefly, PDA NPs were obtained by oxidizing dopamine hydrochloride at room temperature for 24 h in a solution containing alcohol and ammonia. Transmission electron microscopy (TEM) images in Figure 1A demonstrated that the average size of PDA NPs was nearly 75 nm. The FT-IR results showed that the resonance absorption of the C=C bond on the aromatic ring was at 1,635 cm^−1^ suggesting the successful synthesis of PDA NPs (Figure 1B). The cell membrane was extracted from 4T1 cells by differential centrifugation. Then, CuB was loaded into the 4T1 cell membrane by nanoprecipitation. The mixture was extruded repeatedly 15 times through a 400 nm polycarbonate microporous filter membrane to obtain CuB loaded cell membrane (MB) (Supplementary Figure S1). Finally, the MB and PDA NPs were co-extruded through 200 nm polycarbonate microporous filter membrane 15 times to obtain PDA@MB. TEM images demonstrated that PDA@MB NPs negatively stained with phosphotungstic acid had a core-shell structure (Figure 1C; Supplementary Figure S2). The diameter of the PDA core was nearly 75 nm, and the thickness of the outer-membrane shell was nearly 10 nm, which verified the successful combination of MB and PDA NPs. As shown in the UV-vis absorption spectrum (Figure 1D), the characteristic peaks at 228 nm (CuB) and 260 nm (M) are displayed in PDA@MB, indicating that PDA NPs were successfully coated with MB. To examine the existence of membrane (M) proteins on the shell of membrane-biomimetic nanoparticles, sodium dodecyl sulfate polyacrylamide gel electrophoresis (SDS-PAGE) was performed as previously reported (Min et al., 2019). As shown in (Figure 1E), the membrane proteins on PDA@MB could be well retained. The polydispersity index (PDI) of PDA and PDA@MB nanoparticles was .105 and .284, respectively. The changes in hydrodynamic diameter (Figure 1F) indicated the successful synthesis of PDA@MB. After coating with the MB, the zeta potential (Figure 1G) of PDA NPs changed from −18.9 mV to −25.4 mV, which was comparable with that of natural cell membrane vesicles (Supplementary Figure S3), thereby confirming successful coating with the MB. The drug-releasing ability of PDA @MB was tested at pH 5.0 and 7.4. Within 24 h, PDA@MB released 44.64% of CuB at pH 5.0, and 36.11% at pH 7.4 (Supplementary Figure S4A). In contrast, PDA@MB irradiated with NIR laser exhibited 64.57% release over the 24 h (Supplementary Figure S4B). The results showed that both acid and laser irradiation could promote CuB release, but the latter was better.

### 3.2 Photothermal Performance of the PDA@MB

**FIGURE 2:**
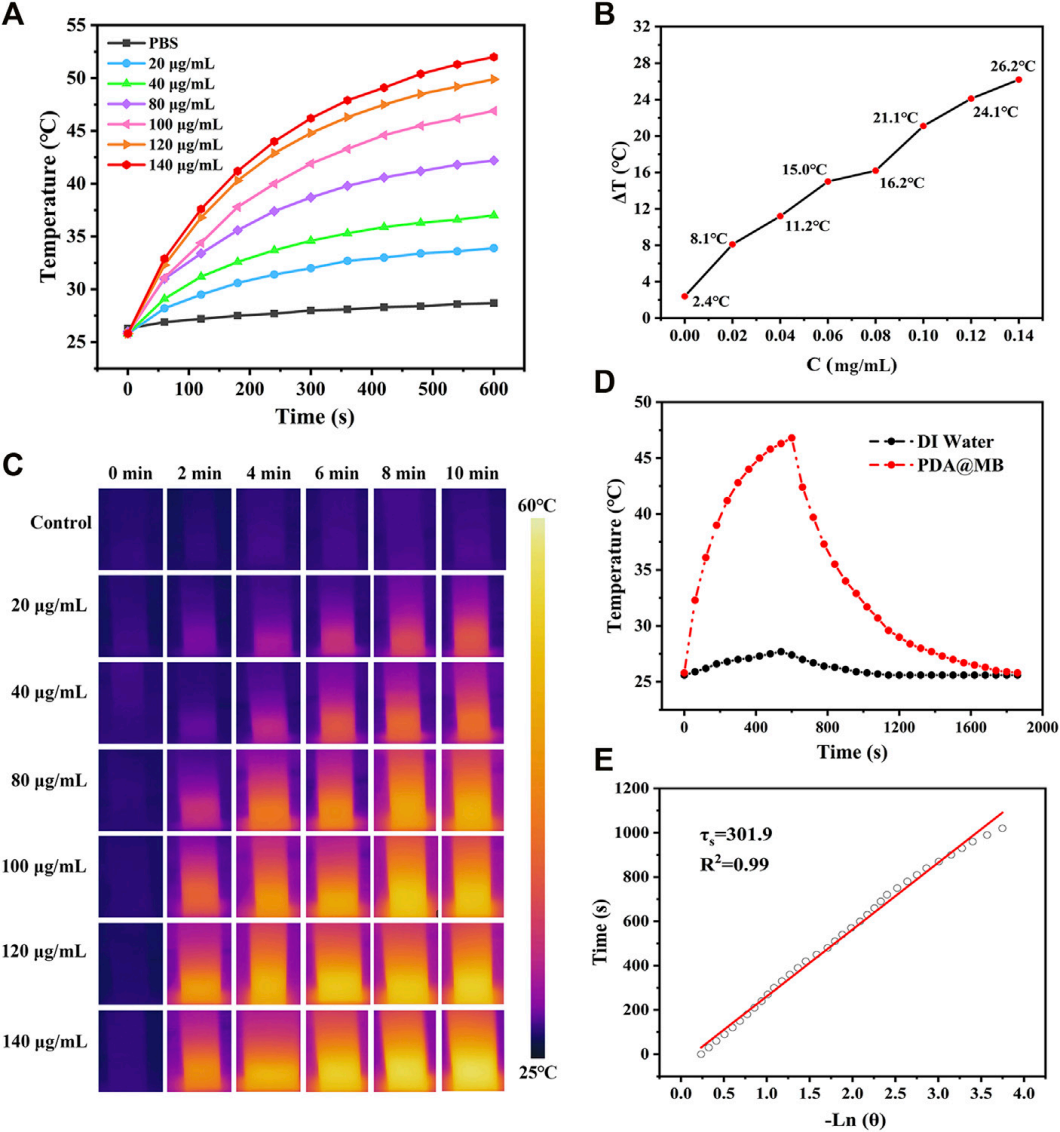
**(A)** Photothermal heating curves of PDA@MB solutions at different concentrations (PDA, 0, 20, 40, 80, 100, 120, and 140 μg/ml) under a 808 nm laser irradiation at a power density of 2 W cm^−2^. **(B)** The temperature variation (ΔT) of PDA@MB at different concentrations upon laser irradiation for 10 min **(C)** IR thermal images of PDA@MB suspensions during NIR laser irradiation. **(D)** Heating and cooling curves of the PDA@MB suspension (100 μg/ml) and deionized water. **(E)** The curve between the cooling time and the negative natural logarithm of the temperature driving force.

The PDA@MB displayed absorbance in the NIR-I biowindow, which exhibited the potential to act as an effective NIR-I PTT photosensitizer. The photothermal conversion effect of the PDA@MB was investigated with different concentrations. Gradient concentrations of PDA@MB suspensions (20, 40, 80, 100, 120 and 140 μg/ml) were exposed to an 808 nm laser with an output of 2 W cm^−2^ for 10 min. As shown in Figures 2A,B, the temperature increased with the irradiation time and concentration. When the concentration reached 140 μg/ml, the temperature increased to 52°C after 808 nm laser irradiation for 10 min. As a control, the temperature of PBS only increased to 28.7°C. The infrared thermal imaging of PDA@MB can be used to identify the location of PTT agent and provide real-time monitoring of PTT (Figure 2C). When the temperature rises to 42°C∼47°C, it will rupture the cell membrane and promote loaded drug release. Therefore, the photothermal performance of PDA@MB could thermally ablate tumor cells, and the rupture of the envelope cell membrane caused by the high temperature could mediate the encapsulated drugs to realize chemotherapy.

Due to the great photothermal performance, we measured the photothermal conversion efficiency (η) of PDA@MB using the method reported in the literature (Liu et al., 2013). The η value was calculated as follows:

$$\eta =\frac{\;h\;A\Delta T_{\max}-Q_{s}}{I\;\left(1-10^{-A_{\lambda }}\right)}$$

where h is the heat transfer coefficient, A is the surface area of the container, ΔT_max_ is the temperature change of the PDA@MB solution at the maximum steady-state temperature, Q_s_ is the heat associated with the light absorbance of the solvent, I is the laser power, A_λ_ is the absorbance of PDA@MB at 808 nm in the UV-vis-NIR spectrum, and η is the photothermal conversion efficiency. According to the obtained data presented in Figures 2D,E, the η of PDA@MB was calculated to be 35.80%. It was even superior to those PTAs including Au anocrystals (21.0%) (Zeng et al., 2013), graphene quantum dots (28.58%) (Cao et al., 2017) and Ti_3_C_2_ nanosheets (30.6%) (Lin et al., 2017). To evaluate the photothermal stability, the PDA@MB solution was irradiated with a 808 nm laser (2 W cm^−2^) for 10 min (laser on) and then the laser was switched off to cool naturally to room temperature (laser off), the cycle was repeated five times (Supplementary Figure S5). The photothermal performance did not display obvious deterioration during the recycling, which suggested the reproducibility and good stability of the nanoagent. These results verify that PDA@MB have potential to induce photothermal ablation of tumor cells.

### 3.3 *In Vitro* Cellular Uptake in 4T1 Cells and RAW264.7 Cells

**FIGURE 3:**
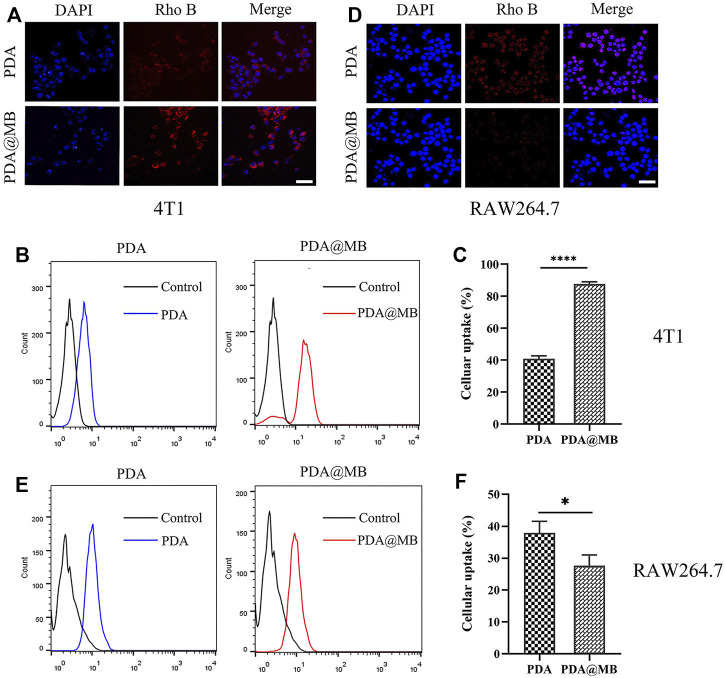
**(A)** CLSM images of 4T1 cells incubated with PDA and PDA@MB. The scale bar is 50 μm. **(B)** Flow cytometric profiles of 4T1 cells incubated with PDA and PDA@MB for 1 h. **(C)** The uptake efficiency of PDA and PDA@MB in 4T1 cells. **(D)** CLSM images of RAW264.7 cells incubated with PDA and PDA@MB. The scale bar is 50 μm. **(E)** Flow cytometric profiles of RAW264.7 cells incubated with PDA and PDA@MB for 1 h. **(F)** The uptake efficiency of PDA and PDA@MB in RAW264.7 cells. Data are represented as mean ± SD (*n* = 3, ∗*p* < .05; ∗∗*p* < .01; ∗∗∗*p* < .001; ∗∗∗∗*p* < .0001).

The cellular uptake studies were performed by confocal laser scanning microscope (CLSM) and flow cytometry. PDA and PDA@MB were labeled with Rho B to indicate the behavior of nanoparticles. The CLSM results are shown in Figure 3A, the Rho B fluorescence in PDA NPs treated 4T1 cells is lower than that of PDA@MB NPs group, demonstrating that the 4T1 cell membrane coating improved cellular uptake in 4T1 cells. This could be due to attributed to the proteins on the cell membrane (Rao et al., 2016). Flow cytometry was used to quantitatively assess cellular uptake. As shown in Figures 3B,C, the cellular uptake of PDA@MB was as high as 87.57% in 4T1 cells, and the fluorescence signal of PDA@MB group is 2.14 times that of PDA NPs group. These results indicated that the cell membrane-biomimetic nanoparticles have high affinity with homologous tumor cells and could increase their cellular uptake.

Next, RAW264.7 cells were used to verify the ability of cell membrane-coated PDA@MB to escape the phagocytosis of macrophages. As shown in Figure 3D, the internalization of PDA@MB NPs is lower than that of PDA NPs in RAW264.7 cells. For flow cytometry quantitative analysis (Figures 3E,F), the fluorescence intensity of PDA NPs group is 1.37 times that of PDA@MB NPs group. These results suggested that the cell membrane-coated PDA@MB could avoid the phagocytosis of macrophages to some extent.

### 3.4 Cellular ROS Assessment and Western Blotting

**FIGURE 4:**
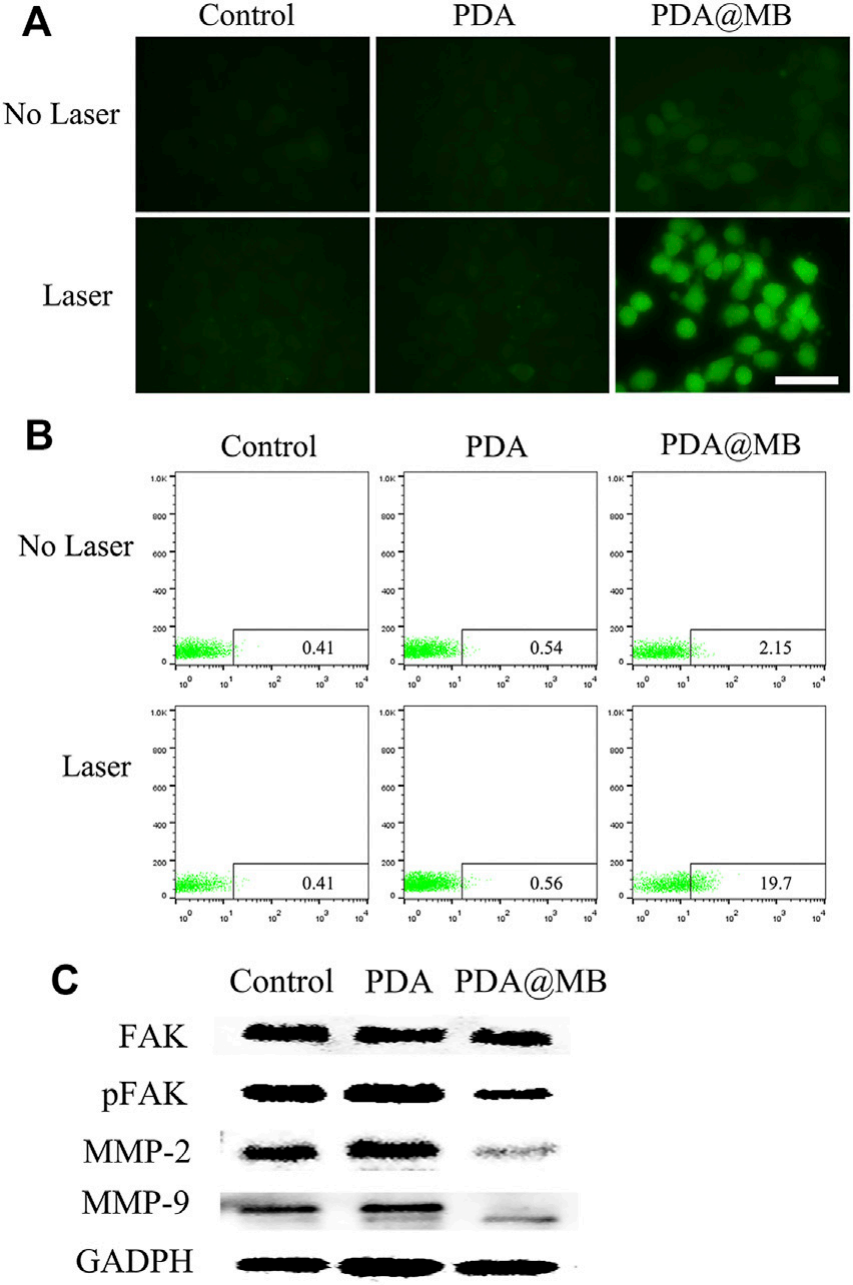
**(A)** CLSM images of 4T1 cells after incubation with PDA and PDA@MB in the absence or presence of NIR by DCFH-DA staining. The scale bar is 50 μm. **(B)** Quantitative flow cytometry results. **(C)** The effect of Control, PDA, and PDA@MB on the expression levels of FAK/MMP signaling pathway proteins.

CuB inhibits the proliferation and metastasis of tumor cells by generating ROS and down-regulating the FAK/MMP signaling pathway (Luo et al., 2018). CLSM and flow cytometry were used to detect DCFH-DA stained ROS. As shown in Figure 4A, almost no green fluorescence is generated without laser irradiation. After NIR laser irradiation, obvious green fluorescence appeared in PDA@MB treated 4T1 cells. This result may be due to the increased uptake of PDA@MB by homologous targeting and the rupture of membrane vesicles under NIR laser irradiation. Thus, the massive release of CuB increased the ROS content. The quantitative detection of ROS production using flow cytometry is shown in Figure 4B. The results also showed that laser irradiation promoted the release of CuB and thus increased the amount of ROS. Previous studies have shown that CuB inhibits the growth of breast cancer cells (Gupta and Srivastava, 2014; Cai et al., 2015; Sinha et al., 2016). It is reported that FAK activation can induce cell invasion by up-regulaing MMP-2 and MMP-9 expression in tumor cells (Schlaepfer et al., 2004). To evaluate the FAK/MMP signaling pathway suppression caused by PDA@MB, the expression of FAK, pFAK, MMP-9 and MMP-2 in cell lysates were detected. As shown in Figure 4C, after laser irradiation, compared with the control group and PDA group, the PDA@MB group significantly inhibited the expression of pFAK, MMP-9 and MMP-2. To further investigate the antimigratory effect of PDA@MB, 4T1 cells were treated with PDA@MB for 24 h and wound healing assays were performed. As shown in Supplementary Figure S6, PDA@MB treated group significantly changed cell morphology, reduced cell viability and inhibited the migratory potential of 4T1 cells under laser irradiation. These above results indicated that the CuB in PDA@MB could improve the hypoxic environment at the tumor site, reduce cell viability, and inhibit the migration ability.

### 3.5 *In Vitro* Anti-Tumor Effect of PDA@MB

**FIGURE 5:**
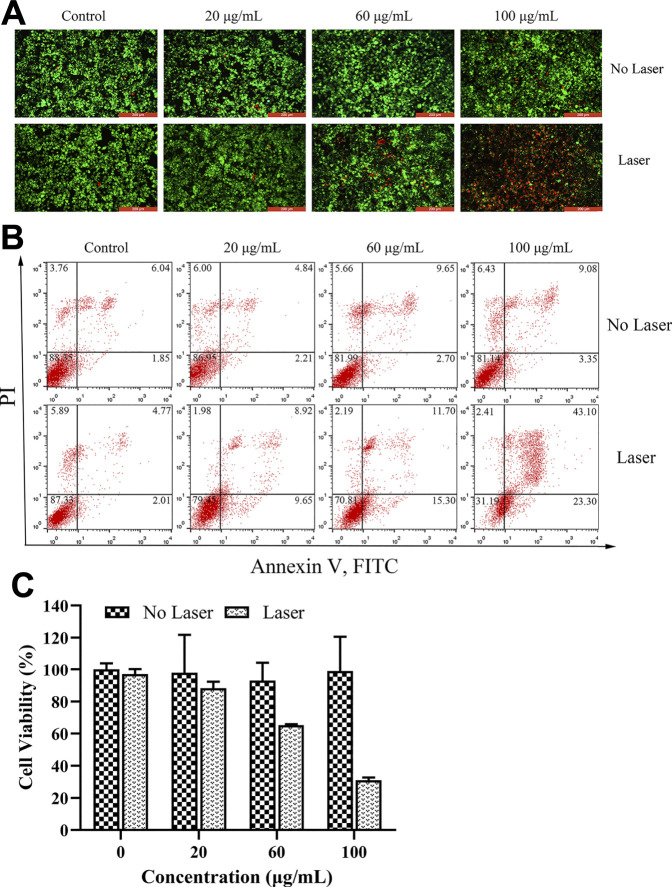
**(A)** Fluorescence images of 4T1 cells upon NIR irradiation (0 and 5 min) with different concentrations of PDA@MB (0, 20, 60, and 100 μg/ml). Green: Calcein-AM, live cells; Red: PI, dead cells; The scale bar is 200 μm. **(B)** Flow cytometric analysis of 4T1 cell apoptosis after treatment with PDA@MB. **(C)** Cell viability of 4T1 cells after treatment with various concentrations of PDA@MB. Data are represented as mean ± SD (*n* = 3).

The *in vitro* anti-tumor effect of PDA@MB was evaluated by Inverted fluorescence microscope, flow cytometry and CCK-8 method. 4T1 cells were incubated with different concentrations of PDA@MB (0, 20, 60, 100 μg/ml PDA, 0, 1.5, 4.5, 7.5 μg/ml CuB). After 5 min of laser irradiation or without laser irradiation, the survival of the cells was detected using the Calcein-AM/PI double staining kit. Live cells are stained green (AM) and dead cells are stained red (PI). As is shown in Figure 5A. Without laser irradiation, 4T1 cells showed clear and vivid green fluorescence at different concentration of PDA@MB, suggesting that they will not be affected by nanoparticles. In contrast, as the concentration of PDA@MB NPs increased, 4T1 cells showed more and more obvious red fluorescence with laser radiation. The quantitative analysis results of flow cytometry are shown in Figure 5B, The concentration of PDA@MB increased from 60 μg/ml (4.5 μg/ml, CuB) to 100 μg/ml (7.5 μg/ml, CuB), the apoptosis rate increased from 27.00% to 66.40%. The cell viability result obtained from CCK-8 assay indicated that the cell viabilities decreased in a dose-dependent manner under NIR laser irradiation (Figure 5C). In addition, the PTT efficacy of PDA, PDA@M, and PDA@MB (100 μg/ml PDA, 7.5 μg/ml CuB) was further evaluated. The results (Supplementary Figure S7) showed that the cell survival rate of the PDA group was 60.00% ± 2.66% under NIR laser irradiation. In contrast, PDA@M and PDA@MB were 39.11% ± 2.43% and 36.14% ± 2.33%, respectively. The results showed that the membrane-modified PDA NPs increased the uptake of 4T1 cells, and the photothermal effect was more significant. These findings demonstrated that PDA@MB hold great promise as an effective PTT agent for tumor therapy.

### 3.6 *In Vivo* Combined Anti-Tumor Effect of PDA@MB

**FIGURE 6:**
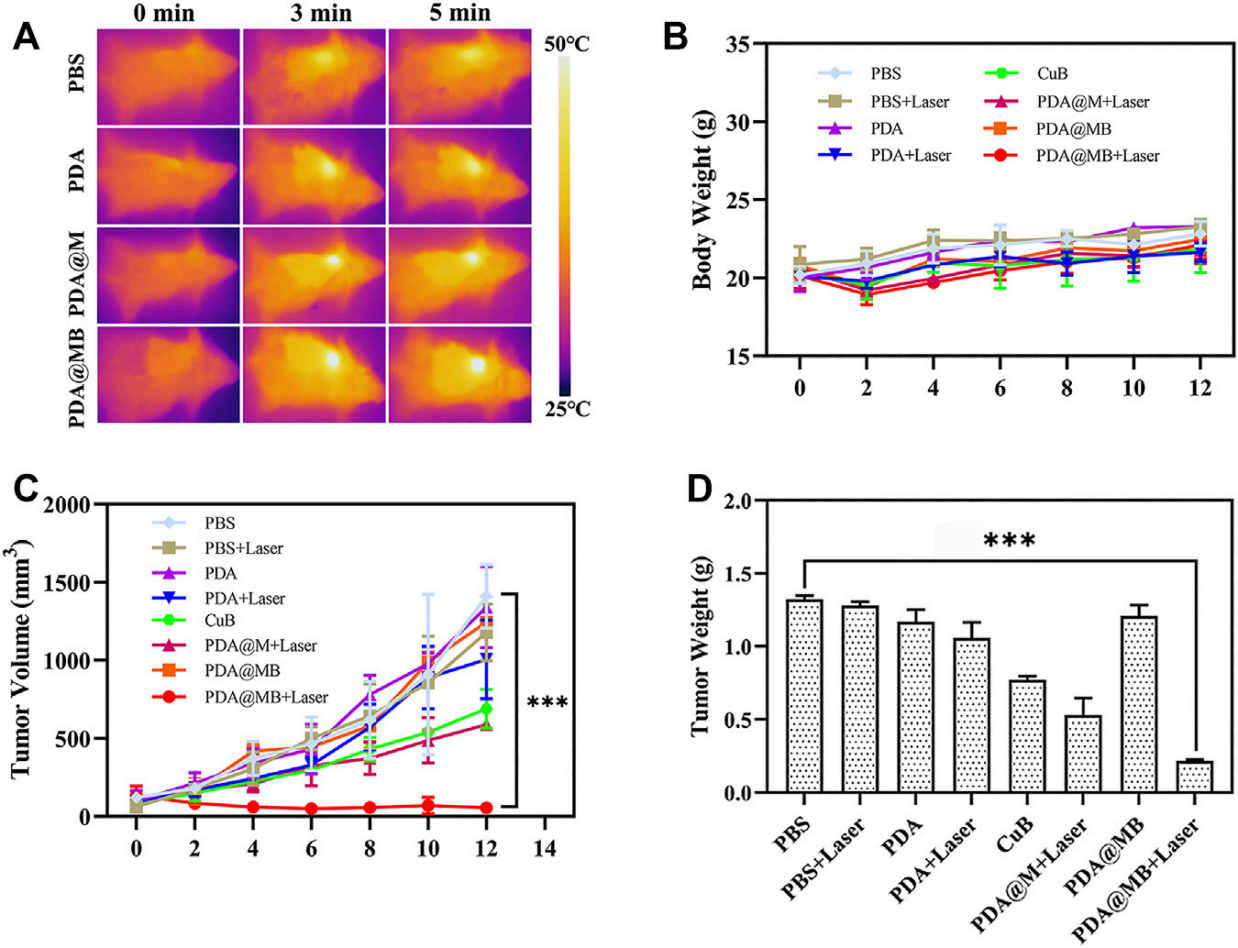
**(A)** IR thermal images of tumors in BALB/c mice during NIR laser irradiation (808 nm, 2 W cm^−2^) after injection with PBS, PDA, CuB, PDA@M, and PDA@MB. **(B)** Body weight curves of BALB/c mice during treatment (*n* = 4). **(C)** Tumor volume curve of BALB/c mice during treatment. **(D)** Average weight of tumors collected from the BALB/c mice after treatments on the 14th day (*n* = 4, mean ± SD, ∗∗*p* < .01, ∗∗∗*p* < .001).

A 4T1 mouse tumor model was constructed to evaluate the combined anti-tumor effect of PDA@MB *in vivo*. On the first day, PBS, PDA, CuB, PDA@M, and PDA@MB (10 mg/kg of PDA, 0.15 mg/kg of CuB, 100 μl) were injected intravenously into mice. The tumor sites of the mice were irradiated with an NIR laser for 5 min (808 nm, 2 W cm ^−2^) and photographed using an IR thermal camera 24 h post-administration. In Figure 6A, the high-contrast real-time infrared thermal imaging results intuitively exhibited the precise tumor targeting and PTT capabilities of PDA@MB. Compared with the PBS and PDA groups, the tumor area temperature in the PDA@M and PDA@MB groups increased significantly, indicating the homologous targeting ability of PDA@M and PDA@MB. After treatment, the tumor size of mice in different groups were measured every 2 days for 14 days. As shown in Figure 6C, the tumor size in the PBS, PBS + Laser, PDA, and PDA@MB groups showed a natural growth trend, suggesting that laser irradiation alone or nanomaterials alone cannot inhibit tumor growth. CuB alone slightly inhibited tumor growth. Compared with the PDA + Laser group, PDA@M + Laser group had a stronger ability to inhibit tumor growth. This may be attributed to the fact that the cell membrane modification increases the uptake of nanoparticles by tumor cells and thus improves the PTT effect. PDA@MB + Laser group had the best effect in all treatments. The tumors in the PDA@MB + Laser group were effectively inhibited at the beginning of the experiment. Such a gratifying result may be due to the release of CuB caused by laser irradiation, thereby down-regulating the FAK/MMP signaling pathway, enhancing the PTT effect of PDA@MB combined with chemotherapy and completely suppressing tumors. Figure 6D shows the tumor mass of mice in each group and Supplementary Figure S8 is the picture of representative mice and tumors after treatment in each group. Consistent with the above results, the mice in the PDA@MB + Laser group showed the lightest tumor mass. Moreover, tumor metastasis in all groups except the PDA@MB + laser group was observed in the liver (marked with red outline) in Supplementary Figure S9. The above results proved that PDA@MB-mediated tumor-targeted combination therapy could provide a new method for cancer treatment.

### 3.7 Toxicology Analysis

Good biocompatibility and low toxicity are essential for nanoparticles in cancer therapy (Ilinskaya and Dobrovolskaia, 2013). The CCK-8 method was used to evaluate the cytotoxicity of PDA@MB in 293T cells (human kidney cells). As shown in Supplementary Figure S10A, the cell survival rate is more than 90% after 24 h of incubation with PDA and PDA@MB. The result of hemolysis assay showed that the hemolysis rate of nanomaterials is less than 5% (Supplementary Figure S10B). These results indicated that PDA@MB had good biological safety and could further conduct anti-cancer research.

**FIGURE 7:**
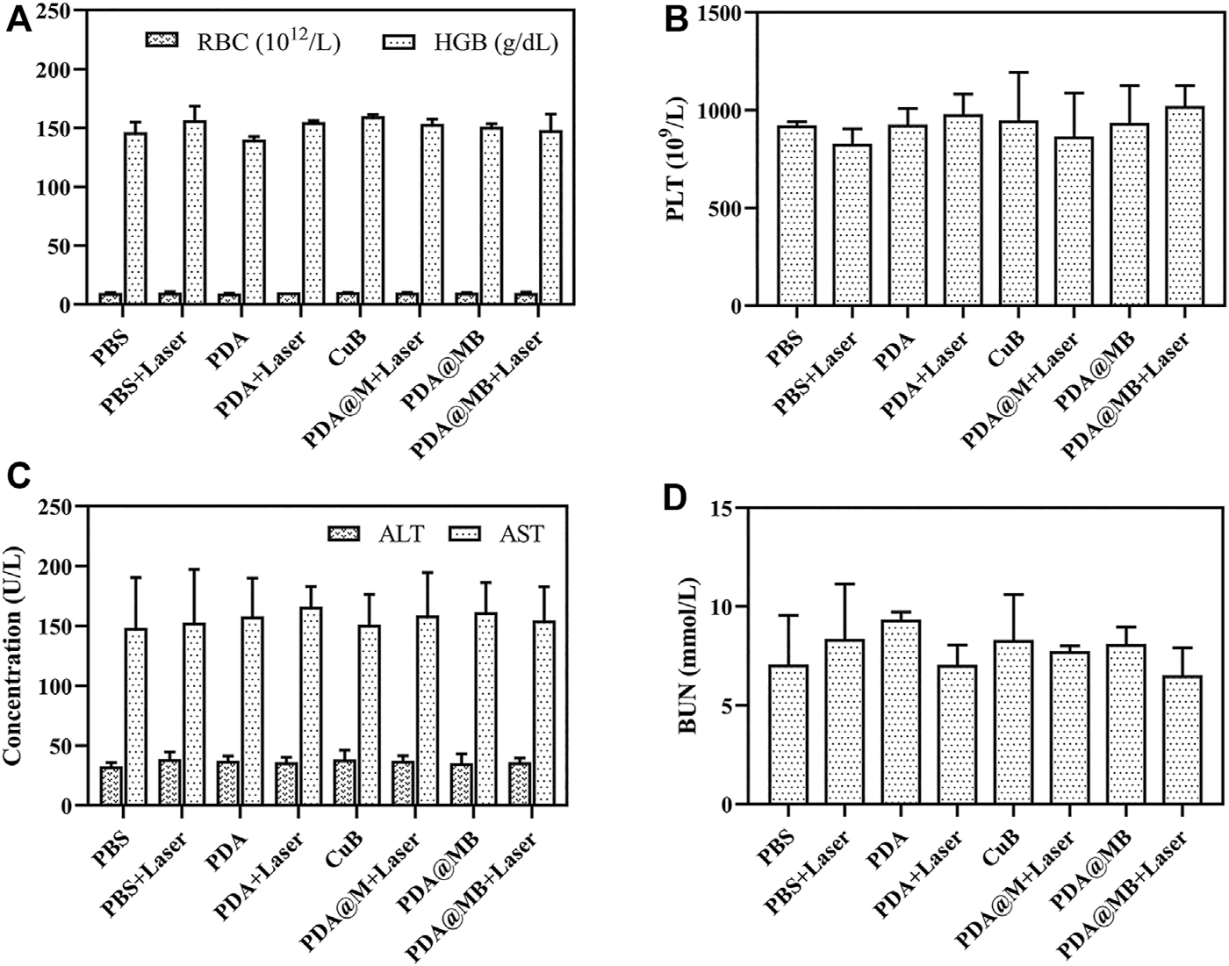
**(A)** Red blood cell (RBC) and hemoglobin (HGB), and **(B)** platelets (PLT) of blood routine examination. **(C)** Alanine aminotransferase (ALT) and aspartate aminotransferase (AST), and **(D)** blood urea nitrogen (BUN) of blood biochemistry test.

The toxic effects of intravenous injection of PDA@MB were evaluated. The body weight of mice treated with nanoparticles was recorded (Figure 6B). Compared with PBS group, the body weight of mice in PBS + Laser, PDA, CuB and PDA@MB groups remained stable, while the body weight of mice in PDA + Laser, PDA@MB + Laser and PDA@MB + Laser groups showed an increasing trend. The weight of mice in all groups did not decrease, indicating that the nanoparticles have good safety. During the treatment period, the eating, activity and excretion of the mice were not affected. In addition, blood biochemistry test, blood routine analysis, and H&E staining examination of major organs (heart, liver, spleen, lung, kidney) were conducted after the treatment. The results of routine blood tests (Figures 7A,B) showed no significant differences in major blood parameters, such as RBC, HGB and PLT. The result proved the good biocompatibility of PDA@MB. The liver injury markers (AST, ALT) and kidney injury index (BUN) of mice in each treatment group were all within the safe range, indicating that each treatment group had no effect on kidney or liver function from the results of biochemical examinations (Figures 7C,D). Finally, the results of histological analysis (Supplementary Figure S9) showed that the main organs of the mice in each group retained their normal physiological morphology. This result suggested that the nanomaterials at our test doses showed almost no or low toxicity *in vivo*.

## 4 Conclusion

In this study, a bionic nano drug delivery platform (PDA@MB) was designed for CPT therapy of breast cancer. The obtained PDA@MB exhibited good biocompatibility, stability, homologous tumor targeting ability and the ability to escape macrophage phagocytosis. The release of CuB from PDA@MB showed photo-responsive release behavior. With high photothermal conversion efficiency (35.80%) and enhanced photothermal therapy in conjunction with chemotherapy, PDA@MB could effectively inhibit tumor growth under NIR laser irradiation. *In vivo* results showed that the tumor mass in the PDA@MB plus laser irradiation group was only .21 g. Moreover, PDA@MB showed no/low toxicity *in vivo* and *in vitro* experiments. In summary, PDA@MB demonstrated an extraordinary combination of chemotherapy and PTT therapy, and may be a promising nano-delivery system for the treatment of cancer.

## Data Availability

The original contributions presented in the study are included in the article/Supplementary Material, further inquiries can be directed to the corresponding author.
