# Supplementary material for: Biomimetic Cucurbitacin B-Polydopamine Nanoparticles for Synergistic Chemo-Photothermal Therapy of Breast Cancer
Source: Front Bioeng Biotechnol. 2022 Feb 9;10:841186. doi: 10.3389/fbioe.2022.841186 (PMC8864241; doi:10.3389/fbioe.2022.841186)
Supplement: Supplementary file 1 [file DataSheet1.docx]

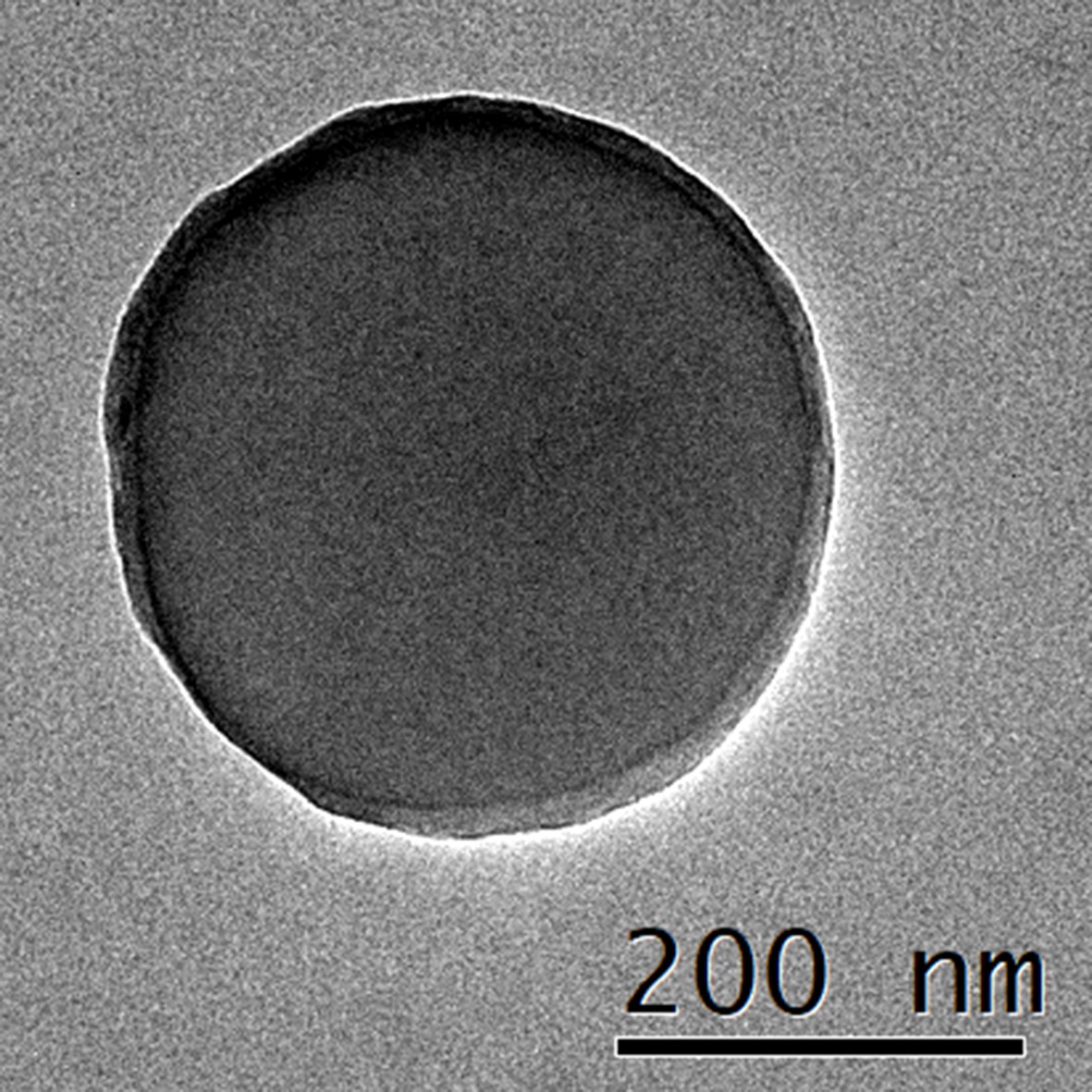


**Suppleme****ntary Figure 1.** TEM images of the CuB loaded cell membrane (MB)


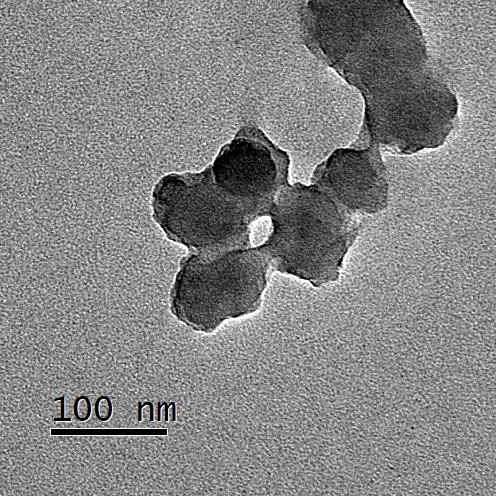


**Supplementary Figure 2.** TEM images of PDA@MB


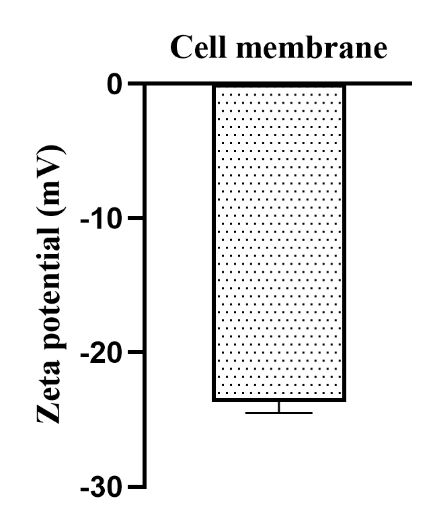


**Supplementary Figure 3.** The zeta potential of 4T1 cell membrane


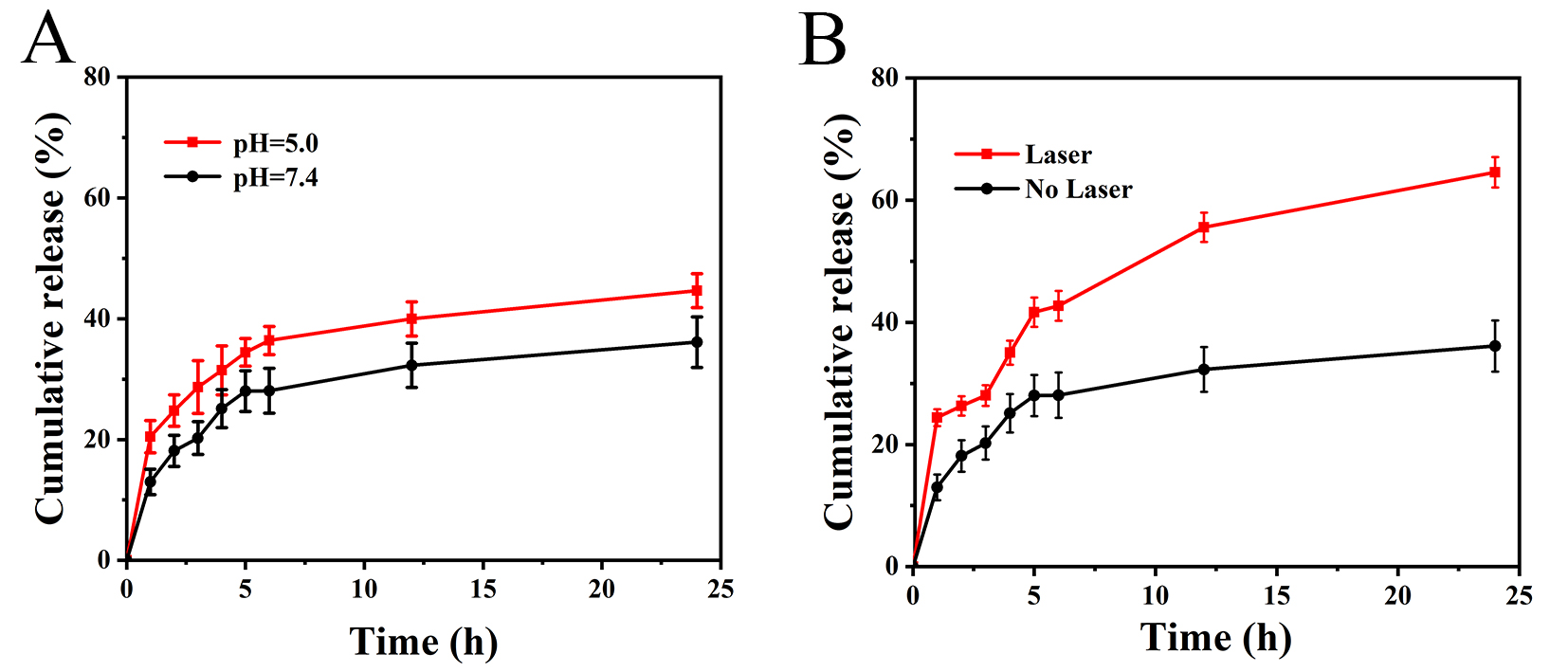


**S****upplementary Figure 4.** (A) In vitro release profiles of CuB from PDA@MB in PBS at pH 7.4 and pH 5.0 at 37 °C. (B) In vitro release profiles of CuB from PDA@MB with or without NIR irradiation in PBS (pH 7.4) at 37 °C.


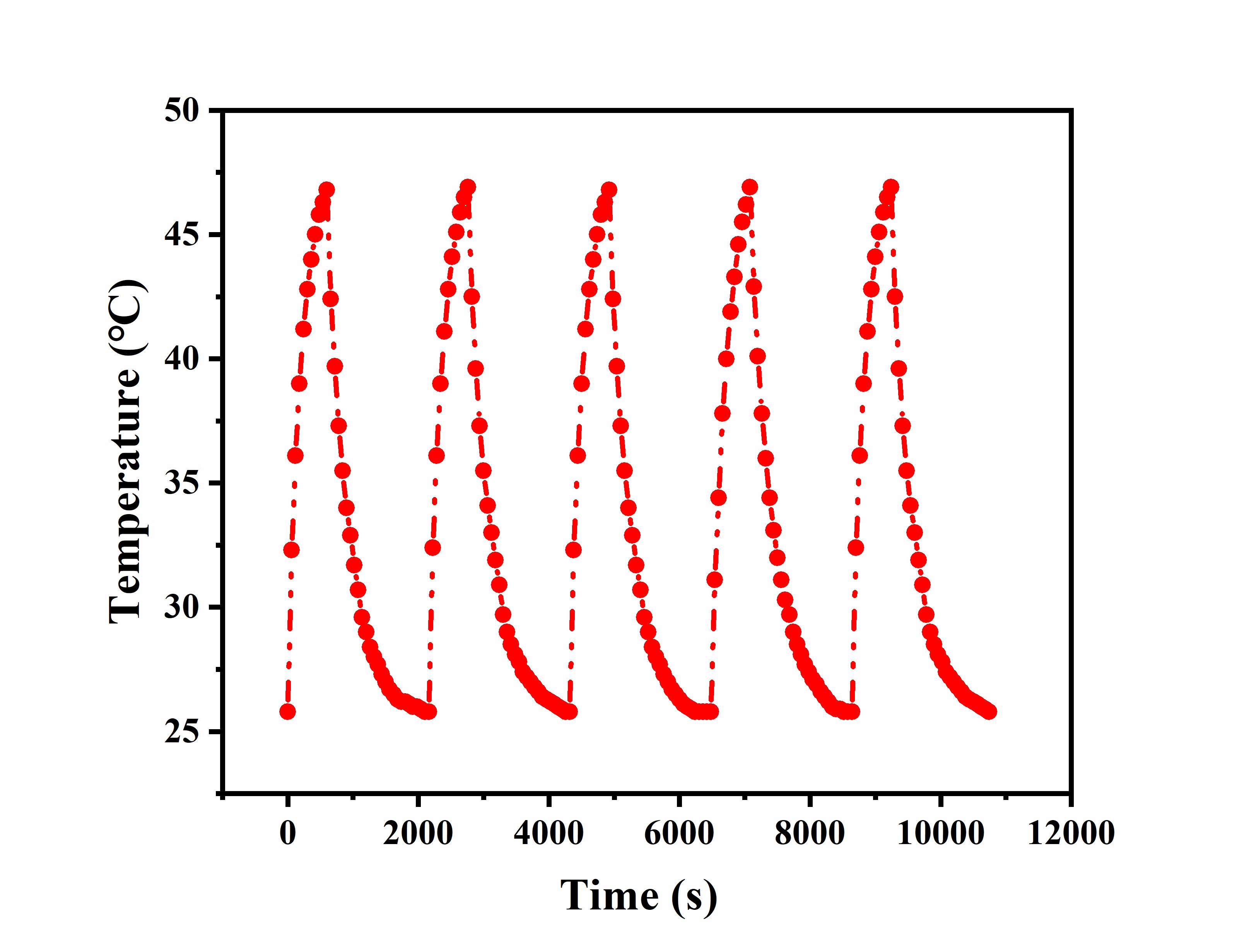


**Supplementary Figure 5.** Photothermal performance of the aqueous dispersion of PDA@MB irradiated with a 808 nm laser (2 W cm^−2^) for 10 min, and then the laser was shut off.


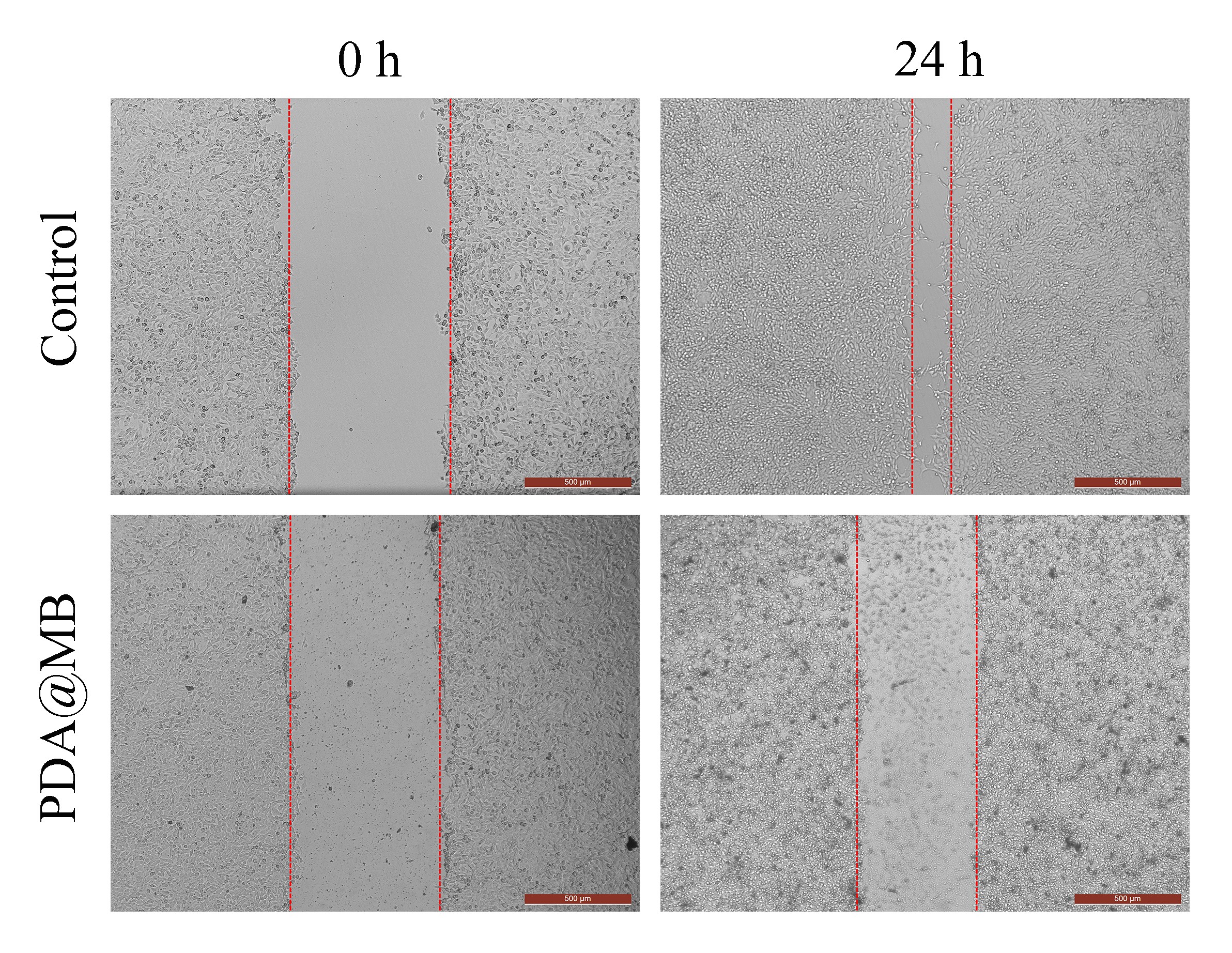


**Supplementary Figure 6.** Typical images of wound healing assessments of control and PDA@MB on 4T1 cells at 0 and 24 h after laser irradiation. The scale bar is 500 μm.


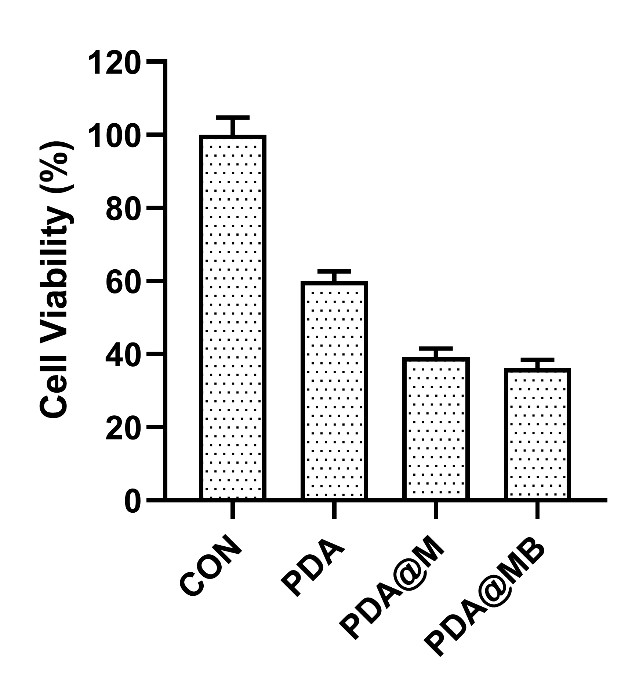


**Supplementary Figure** **7.** Cell viability of 4T1 cells after treatment with PDA, PDA@M or PDA@MB, cells were exposed to 808 nm irradiation at 2 W cm^-2^ for 5 min (n = 3, mean ± SD).


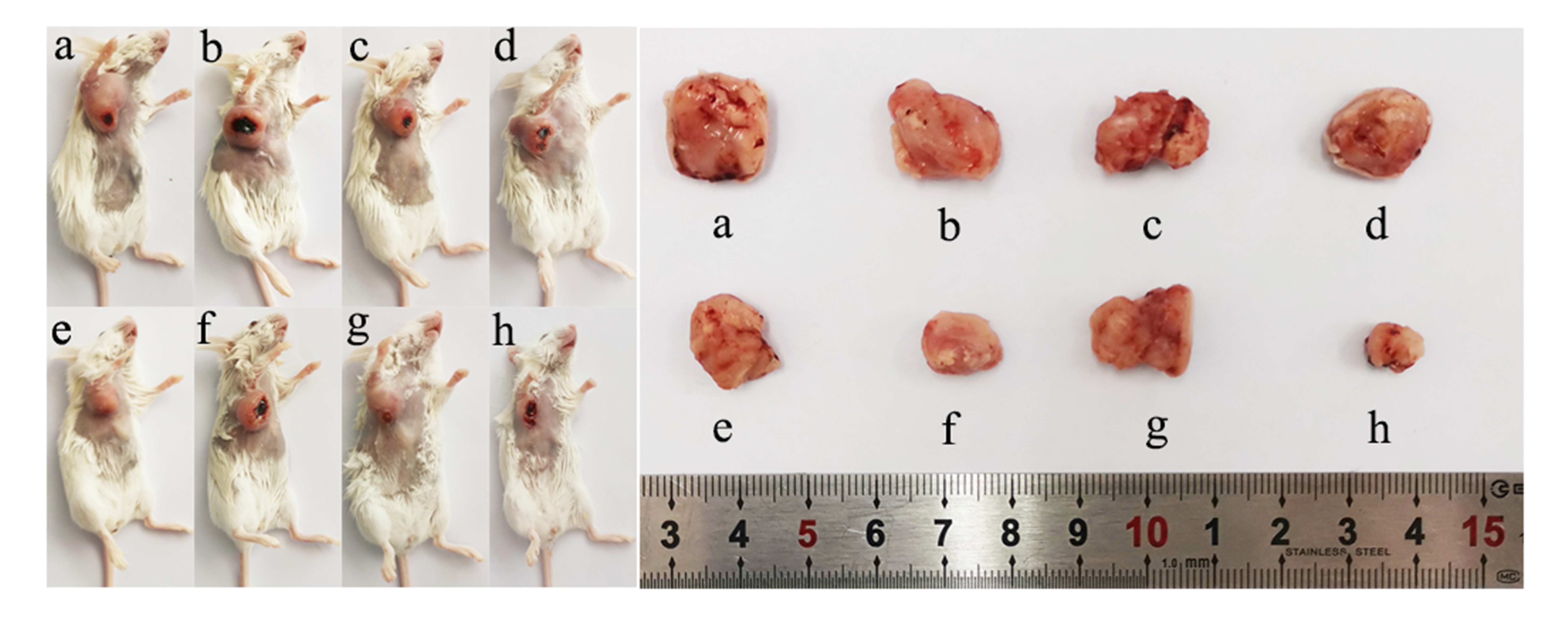


**Supplementary Figure 8.** Representative digital photographs of BALB/c mice and tumors (Group a: PBS; Group b: PBS + Laser; Group c: PDA; Group d: PDA + Laser; Group e: CuB; Group f: PDA@M + Laser; Group g: PDA@MB; Group h: PDA@MB + Laser).


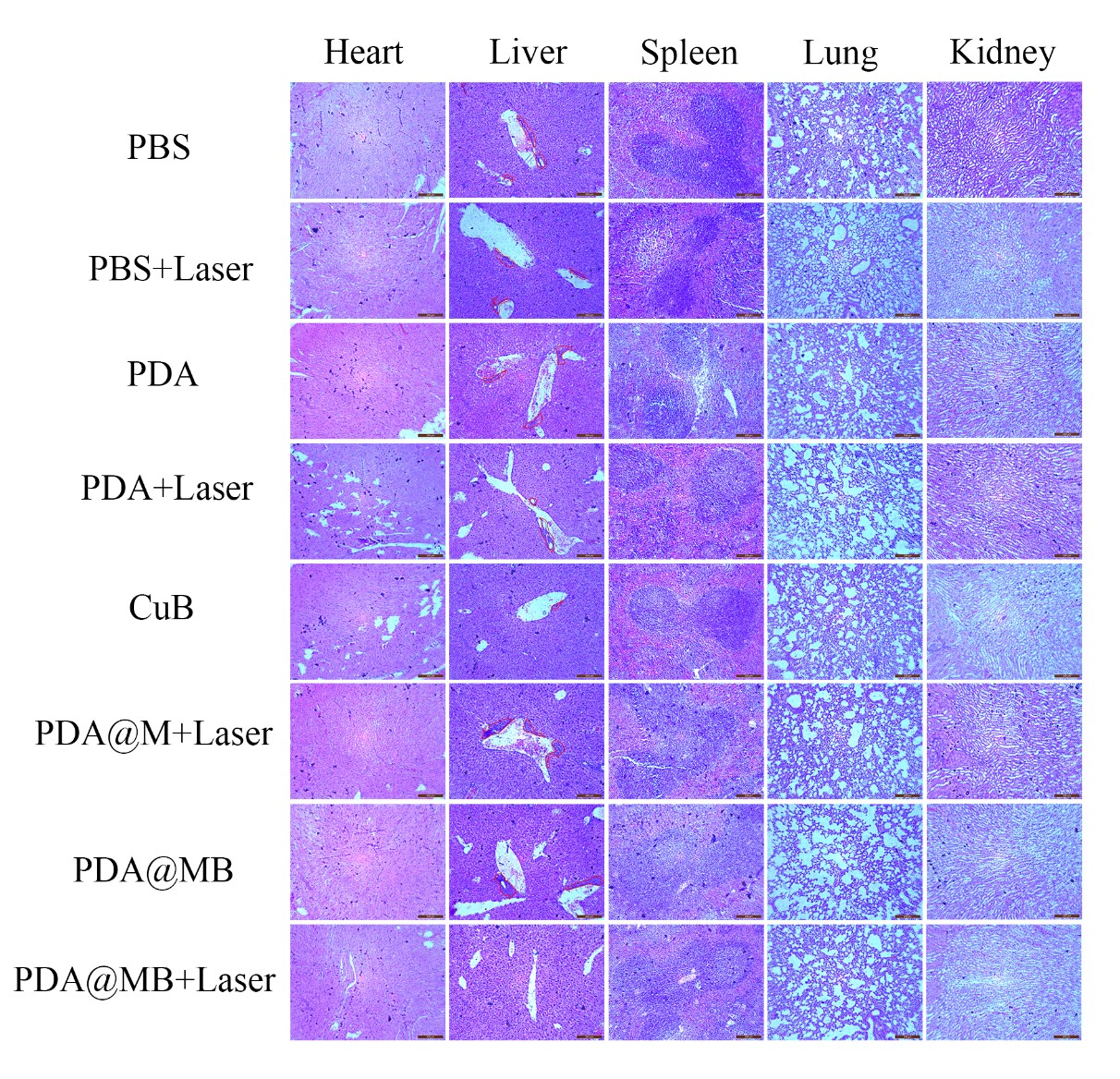


**Supplementary Figure 9.** H&E images of major organs collected from different groups. The scale bar is 200 μm.


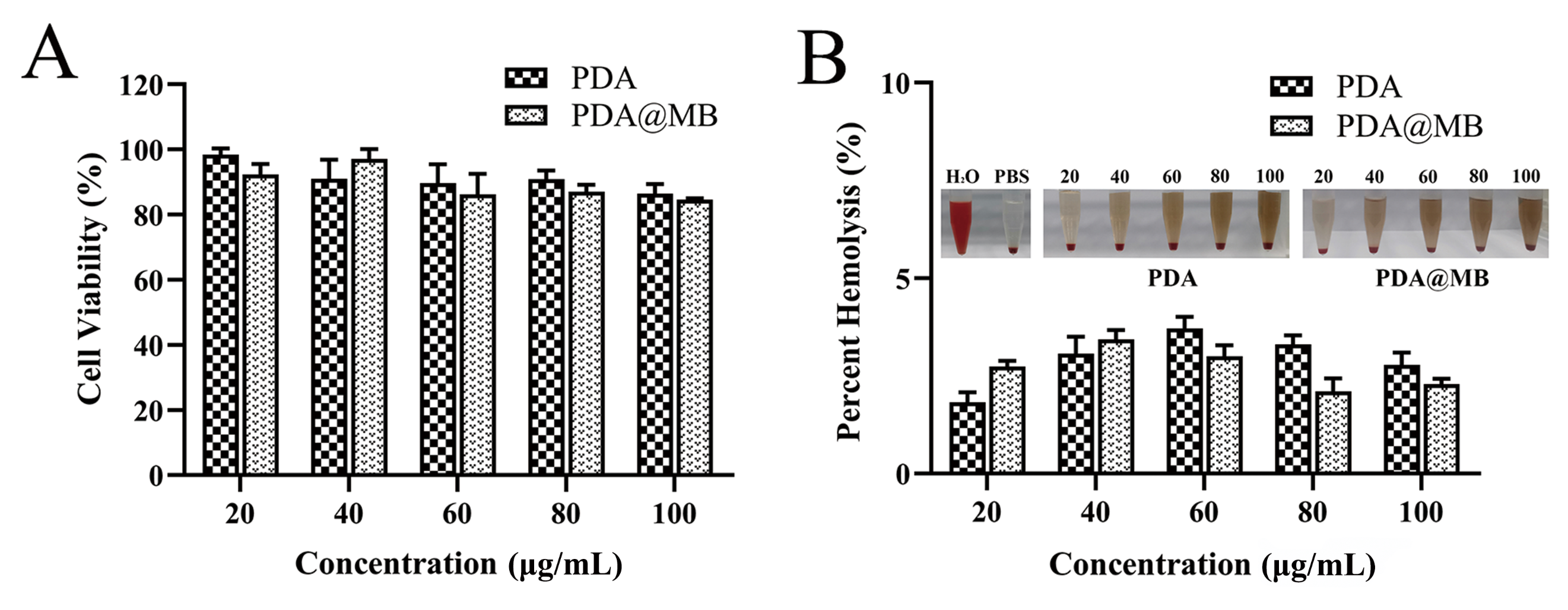


**Supplementary Figure 10. (A)** The 293T cell viability after incubation with PDA and PDA@MB at different concentrations for 24 h. **(B)** Hemolysis of PDA and PDA@MB at different concentrations. Data are represented as mean ± SD (n = 3).­­
